# Extracting and transforming clinical guidelines into pathway models for different hospital information systems

**DOI:** 10.1186/2047-2501-1-13

**Published:** 2013-11-04

**Authors:** Britta Böckmann, Katja Heiden

**Affiliations:** University of Applied Sciences and Arts Dortmund, Emil-Figge-Straße 42, 44227 Dortmund, Germany

**Keywords:** Clinical pathways, Clinical guidelines, Meta-modelling, Health level 7, Ontologies, Hospital information systems

## Abstract

**Background:**

Healthcare providers are facing an enormous cost pressure and a scarcity of resources. They need to realign in the tension between economic efficiency and demand-oriented healthcare. Clinical guidelines and clinical pathways are used in German hospitals to improve the quality of care and to reduce costs at the same time. Clinical guidelines provide evident medical knowledge for diagnostic and therapeutic issues, while clinical pathways are a road map of patient management. The consideration of clinical guidelines during pathway development is highly recommended. But the transfer of evident knowledge (clinical guidelines) to care processes (clinical pathways) is not straightforward due to different information contents and structures.

**Methods:**

We propose a model-based approach to support the development of guideline-compliant pathways and the generation of ready-to-use pathway models for different hospital information systems. A meta-model merges the structures of clinical guidelines and clinical pathways into one generic model. It is encoded through artefacts of Health Level 7 (HL7) in version 3. The deployment process to integrate the defined guideline-compliant pathways into different target systems is supported by an ontology management approach.

**Results:**

We defined a step-by-step instruction for translating the narrative guideline content into formalized care processes. The meta-model provides all necessary structures to capture the pertinent knowledge. The entire process of defining and deploying guideline-compliant pathways is supported by one consistent IT system. The deployment process is designed detached from specific systems so that the defined pathways can be enacted within different hospital information systems (HIS).

**Conclusions:**

The approach enables hospitals to develop guideline-compliant pathways and to integrate them into their HIS without time-consuming manual transformations. That way, best practice advices based on clinical guidelines can be provided at the point of care and therefore improve patient treatment.

## Background

Clinical guidelines and clinical pathways are accepted instruments for the quality assurance and process optimization in the healthcare domain. Both concepts define a standardized best practice about appropriate patient care for a specific disease. Clinical pathways are defined as “a complex intervention for the mutual decision making and organization of care processes for a well-defined group of patients during a well-defined period” [1]. They provide a process-like description of proper medical treatment, whereas clinical guidelines are defined as “systematically developed statements to assist practitioner and patient decisions about appropriate health care for specific clinical circumstances” [2]. They represent results of latest research. The positive impact of clinical guidelines on the quality of care has been scientifically proven in [3]. But their influence on clinical routine is still very low in Germany due to their narrative and non-formalized form [4]. A decisive factor for the success and use of clinical guidelines is the provision of the knowledge at the point of care [5].

The main challenge can be summarized as follows; imprecise, not formalized and abstract guidelines have to be implemented as concrete processes. Clinical pathways are appropriate for that purpose; they are used in different kinds of healthcare facilities, facing a diversity of hospital information systems (HIS). Following, an IT-based approach is presented, which allows the derivation of guideline-compliant pathways and the generation of evidence-based pathway models for different target systems.

## Methods

There exist different approaches to implement guideline recommendations into clinical routine. They show considerable differences concerning the aim or result of the translation process (clinical pathways vs. computer-interpretable guidelines). In addition, they vary in the degree of automation (highly manual vs. semi-automated approaches). We evaluated related work to point out the remaining problems in that research field.

To bridge the gap between clinical guidelines and clinical pathways, we propose a model-driven approach. Thus, the essential part is a meta-model, which aims to integrate information coming from the guidelines and offers pathway structures to formalize the guideline recommendations. It provides all allowed entities, and relationships to describe a guideline-compliant pathway. For the definition of the meta-model, different sources of information were used. The model construction started with the analysis of existing clinical guidelines. First, the guideline for the diagnosis and treatment of breast cancer was used [6]. The aim of this step was to identify general components of a guideline by abstracting from concrete, disease-specific recommendations. In addition, the guideline for asthma [7] and chronic heart failure [8] were regarded to extend and concrete the list of guideline elements. Based on these results, a preliminary meta-model was defined and visualized by an UML class diagram. In addition, we analysed different guideline modelling languages (Asbru, GLIF, and GEM), which were developed to support the computerization of guidelines; cf. [9]. These representation languages already provide necessary elements to formalize the guideline content. The meta-model was extended by further attributes and UML classes, which were gathered by the evaluation of the guideline modelling languages. Beside the guideline structures, the content of clinical pathways should be incorporated into the meta-model. To integrate path-specific structures, we analysed path modules integrated within different HIS as well as the Health Level 7 Care Plan Model [10], a standard to describe clinical pathways. Based on all these findings, the meta-model was constructed and finalized. The encoding is given through structures of Health Level 7 (HL7), a worldwide accepted standard in the healthcare domain. HL7 provides a comprehensive framework for interoperability that improves care delivery, optimizes workflow, reduces ambiguity, and enhances knowledge transfer among all of the stakeholders; see [10]. It ensures a platform independent description of guideline-compliant pathways until they are deployed in concrete information systems. The encoding was done on basis of the HL7 Care Plan Model, which has been developed by the HL7 group to define action plans for various clinical pictures. The HL7 Care Plan Model is not a normative standard yet [10]; rather it is a draft, which can be extended and adapted to depict the meta-model of guideline-compliant pathways.

The deployment process includes the transformation of the defined pathways, described through the structures of the meta-model, into different target formalisms of the HIS. It is realized by an ontology-based approach. Gruber [11] defines an ontology as an “explicit formal specification of a shared conceptualisation”. Ontologies represent a key technology, which enable semantic interoperability and integration of data and process [12]. “Semantic” means the mapping between a language or modelling syntax to some formalism, which expresses the “meaning” [13]. Semantic applications do not focus on the presentation of data rather they focus on the subjects and their relationships. The main advantage is the explicit representation of these concepts, that underlie an application and needed presentations are generated by these specifications [13]. In this approach, different metadata schemes of HIS describing clinical pathways need to be linked. Ontologies are employed to define the component mapping between the elements of the meta-model and the elements of the target systems. This ensures that the system knows about the available facilities of the target system to represent a clinical pathway. Furthermore, the ontologies are used to navigate the domain experts through the deployment process. They should be enabled to realize the configuration of the defined pathways within their target systems. The main advantage of modelling the component mapping ontologically instead of hard coding this information content is the possibility to extend or adapt the knowledge consecutively. If the data model of a target HIS changes, these modifications can be made intuitively. We used the Web Ontology Language (OWL) for the formal representation of the component mappings. To perform the initial mapping, detailed knowledge about the target systems is required. Therefore, a system analysis is done for every target HIS identifying the available components to represent the information contained in the meta-model.

## Related work

Several methodological approaches exist to implement clinical guidelines into operational practice. One approach focus the formalization of narrative guidelines in a computer-interpretable form (see [14, 15]), which can be processed in decision support systems. The domain experts are supported during this process by special editors. It is error-prone to map prose text to coded data, because clinical guidelines can partly be ambiguous, incomplete, or even inconsistent [16]. Several guideline representation languages exist as rule-based languages, e.g. Arden Syntax, logic-based languages, e.g. PROforma, task network models, e.g. Asbru, GLIF, or document-centric approaches i.e. GEM. Differences and similarities of these languages are outlined in [9, 17]. If computer-interpretable guidelines should be used by a hospital in order to provide the medical knowledge during patient care, the hospital information systems need to have the ability to interpret and use those formalizations or the HIS needs to interact with a decision support system. The translation process does not produce a clinical pathway by definition; rather computer-interpretable guidelines (CIGs) are created to support the decision making process during patient treatment. This approach provides one way to implement guideline recommendations in daily routine, but, according to [5], the translation of clinical guidelines into alerts and reminders does not support the patient treatment as a unit.

The second approach is a highly manual process, where clinical pathways are developed on the basis of existing clinical guidelines (see [18–20]). The pathway development process is done by a group of domain experts. An interdisciplinary team is composed of all professional groups involved, e.g. physicians, nurses, medical controllers, quality assurance representatives. At this approach, the pathway development process starts with an extensive literature research, where pertinent guidelines are identified. The analysis needs to be done manually by the interdisciplinary team or a single project member. It is a time-consuming and resource-intensive task with no methodical support. The extracted recommendations from the guidelines can be used as an input for the pathway development. Therefore, the guideline content needs to be tailored to local conditions and a consensus among the participating health professionals needs to be reached [5]. Information technology is mainly used for modelling tasks and not for the whole lifecycle management of clinical pathways. The result of this development process is a clinical pathway for one specific healthcare facility. The interdisciplinary team produces text documents or informal process models, which describe the appropriate care for a specific disease in form of a clinical pathway. Thus, the developed pathways cannot be directly interpreted by IT systems. A formalized description and additional technical information for the enactment of clinical pathways needs to be defined, e.g., mapping of service calls to specific tasks. The implementation of clinical pathways is a separate step, which is done by IT-specialists. The experts often do not have detailed domain knowledge and sometimes the pathway definitions are ambiguous. There is a high need for communication between the domain- and the IT-specialists. Several cycles are necessary to implement the pathways in the present information system. Thus, a gap between development and implementation of clinical pathways exists as well as a media break between both process steps.

A further approach focuses the systematic derivation of clinical pathways from clinical guidelines by the help of a model-based methodology (see [21, 22]). Jacobs [21] developed a reference model for the methodical transfer of clinical guidelines in clinical pathways, which was exemplary deduced from the breast cancer treatment. One universal pathway for a specific guideline was derived, which can be adapted to a special institution in a further step. Jacobs performed a theoretical examination of the derivation process; but no consistent IT support for the implementation of the derived clinical pathways in the present information system was provided. Schlieter [22] carried the results from Jacobs forward. Rule sets were added to the reference model in order to define the way of reusing model content. Schlieter provides construction techniques for deriving specific models from a formalized guideline.

After presenting the related approaches, the following requirements can be imposed, as a quintessence, for our work. In contrast to other approaches, the whole development process (definition, implementation, lifecycle management) is supported. Each presented approach covers only one aspect of the entire derivation process; a formal representation (see CIGs), the development of concrete clinical pathways for one specific institution (manual process), or the systematic translation (model-based approach). The work in hand creates a guideline-compliant clinical pathway, which describe the whole patient treatment of a special disease in one concrete setting. Additionally, ready-to-use path models are generated for different hospital information systems to implement the derived pathways. Related approaches especially neglect the last step (deployment), although it is a decisive factor for the success and usage of clinical pathways.

## Results

### Model-driven approach

**Figure 1:**
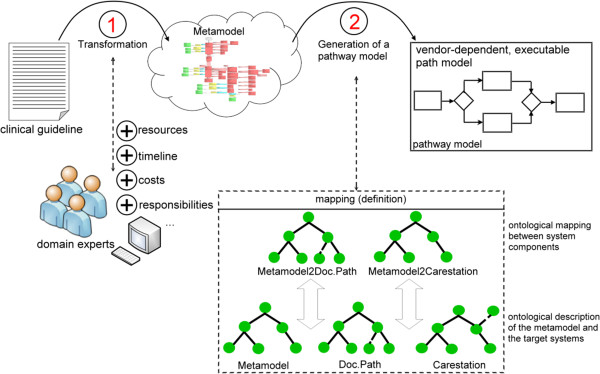
**Model-driven approach.** Illustrates all necessary steps for defining guideline-compliant pathways.

The key part of the model-driven approach is the underlying meta-model, which provides a formalized description of evidence-based pathways and stores all extracted information. A meta-model offers the ability to represent data structures of a special domain in a concise way. Our goal is the provision of a meta-model that underlies both; the knowledge elicitation process (step 1 in Figure 1) and the generation process (step 2 in Figure 1). The entire model-driven approach for defining guideline-compliant pathways is supported by information technology. Therefore it consists, in general, of two different modules; a modelling component to define a clinical pathway on the basis of existing clinical guidelines and a generation component, which supports the deployment of these pathways in concrete information systems. Before we point out, how the derivation of guideline-compliant pathways takes place, we briefly present the meta-model.

### Meta-model

**Figure 2:**
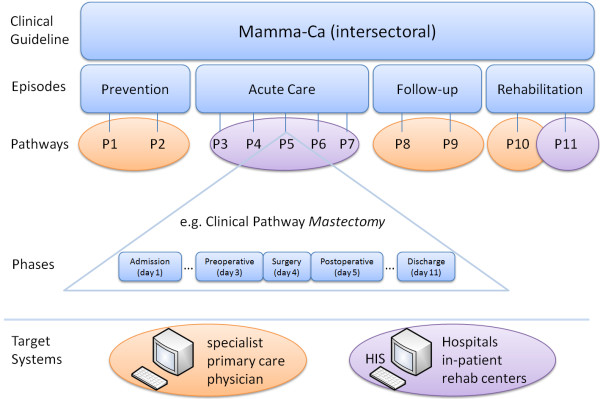
**Structure of the meta-model.** Clarifies the structure of the meta-model using the example of the breast cancer guideline.

From a top-level perspective, our model is able to adopt the concepts of both, clinical guidelines and clinical pathways, to obtain a merged view as a final result. Clinical guidelines often provide recommendations for the whole medical treatment of a disease including different episodes of care, e.g. prevention, diagnostics, therapeutics, follow-up, and rehabilitation. Therefore the model offers elements to describe an intersectoral pathway. Every episode of care can be supported by different healthcare facilities and is represented by a set of clinical pathways. These pathways describe the entire care activities for one episode. Figure 2 exemplary summarizes that; the episode acute care is completely described through the pathways P3 – P7. Each pathway can be structured into different treatment phases, e.g. a preoperative day, a day of surgery, and a postoperative day, see Figure 2. Each phase in turn is described by a set of medical, nursing, and administrative activities. By using HL7, there exist different activity types, which can be used to describe the patient treatment, e.g. procedures, medications, encounters, and observations. The control flow during patient treatment is modelled through *HL7 workflow control suite of attributes.* Responsibilities in the care process and necessary resources are considered by the model as well. Beside these process-like structures, additional information from clinical guidelines, e.g. risk factors, complications, or guiding symptoms, is considered. This information has no influence on the control flow of patient treatment, but it can create added values especially for young professionals and thus should be displayed on demand in the target systems. To depict this information, a generic parameter system has been integrated in the meta-model.

The HL7 Care Plan Model was extended by the following classes to depict the entire elements of the meta-model:

1. Assignment of clinical guidelines to clinical pathway; that way the evident basis for the definition of a guideline-compliant pathway is stored
2. Adding structural components to define intersectoral pathways, and to divide the whole patient treatment in coherent units, e.g. episodes of care
3. Defining concepts like costs, strength of evidence and recommendation, and so on (those contents cannot be depicted by the original model)
4. Integrating the HL7 Control Suite of Attributes to enable the definition of a detailed control flow
5. Representing additional information within a parameter system

### Derivation process

The derivation process of defining guideline compliant-pathways is done by an interdisciplinary team. They use existing (narrative) clinical guidelines and need to perform three different steps.

**Figure 3:**
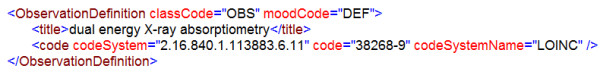
**HL7 representation of a dual energy X-ray absorptiometry.** The XML excerpt shows the description of the X-ray test within the meta-model and a HL7 specific XML notation.

-  Step 1: Extraction of all pertinent guideline recommendations, which should be considered during pathway development, and classification of this information. The classification is done based on the elements of the meta-model and indicates the content of a narrative recommendation, e.g. medication, procedure, observation, additional information. This step is supported by an integrated mark-up tool.
-  Example: The guideline recommendation “Patients with early invasive breast cancer should have a baseline dual energy X-ray absorptiometry (DEXA) scan to assess bone mineral density” [23] is classified as an examination (observation).
-  Step 2: Formalization of the recommendations; through the classification in the previous step, the correlation between the meta-model and the guideline excerpts is already given. Within the meta-model all descriptive attributes for a specific element are defined, e.g. a procedure consists of a name, an OPS code (the German modification of the ICPM), and so on. Based on this definition, customized forms are generated to capture the remaining information in order to formalize the narrative recommendations. This concretization is important to ensure the subsequent enactment and the use of this guideline fragments for pathway definition. The content is not necessarily part of the clinical guideline, e.g. a dosage for a medication is not always specified within the guideline. Missing data can be complemented by the domain experts. Step 2 is completed by adding further activities, which are not defined within clinical guidelines, but which need to be performed during patient treatment, i.e. nursing activities. After step 2 all recommendations are represented in a computer-interpretable form (HL7).
-  The following representation of the DEXA examination is produced by the IT system (see Figure 3). The domain experts do not need any knowledge about the meta-model and its representation. They use generated forms to fill in all necessary data and the system stores the formalized elements within the structures of the meta-model.

-  Step 3: Composition of a guideline-compliant pathway by sequencing the defined activities, by adding responsibilities and resources, by defining a detailed control flow and by adding quantified time information for execution. Step 3 is supported by a graphical editor. The input is the list of all formalized activities (step 2).

The derivation process can be summarized as followed; relevant guideline excerpts are translated into path-specific structures in a step-by-step method. General and nonspecific guideline recommendations for diagnostic and therapeutic activities are specified for the definition of evidence-based pathways. The result of this process is a clinical pathway in a vendor neutral description (meta-model). It bases on evident clinical guidelines and is represented by a worldwide accepted standard in the healthcare domain (HL7). The IT system supports the domain experts in performing the derivation process. The main goal is to hide the complexity of the meta-model and provide an intelligent navigation through the whole process. The domain experts need to have the ability to use the IT system without having knowledge about the underlying model and the target representation of the backend system.

### Generation of ready-to-use pathway models for different hospital information systems

After the derivation process, the guideline-compliant pathways are defined and represented within the meta-model. Most of the healthcare facilities use and manage clinical pathways in their information systems. Therefore the next step is the deployment of the guideline-compliant pathways. The enactment of clinical pathways within a hospital information system can create the most added values, i.e. a continuous surveillance of clinical outcome is possible or routinely documentation tasks can be reduced, because only derivations from a pathway need to be recorded. There exists a diversity of HIS in German hospitals. The HIS vendors use different strategies and formalisms to model and enact clinical pathways. Either they use existing components of the HIS or explicit process models, e.g. Business Process Model and Notation (BPMN), or XML Process Definition Language (XPDL). The guideline-compliant pathways, which are depicted within the meta-model, are described by a HL7 specific XML structure. As a consequence, to deploy the guideline-compliant pathways, they need to be translated into different formalisms of the target systems, i.e. SQL or various XML syntaxes. This should be supported by the IT system as well to avoid time-consuming manual transformations. The deployment process is separated into two different steps; a mapping between the elements of the meta-model and the elements of the target systems needs to be done (component mapping). In addition, a technical translation of the pathway into the target formalism has to be realized. Both steps are explained below.

### Component mapping

The component ontology ensures that every element of the meta-model is mapped to one or more equivalent representations in a target system. It is possible that an element of the meta-model has only one counterpart, e.g. a medication. But it is possible too, that there are several ways to represent an element in a target system. This will be clarified by the following example.

The HIS iMedOne (brightONE) provides the path module DOC.Path to manage and enact clinical pathways. Within DOC.Path the elements of the pathway are scheduled in a calendric view and are assigned to different dimensions. A dimension aggregates equal element types, e.g. orders, medications, nursing activities, so that various views can be defined for different user groups. The element medication in the meta-model has only one equivalent in DOC.Path; a prescription. But additional information out of the generic parameter system could be represented in different ways, e.g. a new dimension *information* can be created and the content can be placed within such an element, or this information is displayed within the textual description of a specific path element, or it is represented by a document or an URL, which can be added to every path element.

Based on the possible representation forms defined within the component mapping, the interdisciplinary path team has the choice, how to implement the guideline-compliant pathway. Again the users are supported by the IT system, because the system knows about the available facilities of the target system and can therefore offer the possible elements to the domain experts, who can perform the configuration of the pathway. As a consequence, the arrangement of the pathway models in a special system is no black box for the domain experts. In contrast, they can influence the results by interacting with the IT system. The arrangement of a pathway in a concrete information system may even vary between different users. Thus, the configuration can be stored individually to adapt the generated suggestions to each user.

Concerning the generation of concrete pathway models, there exist, in general, two different kinds of information; active and passive elements. Passive elements, i.e. additional information, can be simply copied to a target system and can be handled as pure data. In contrast, active elements require the creation of special elements in the target system and therefore the interpretation of the information content. For instance, a lab test described within the meta-model, would lead to a concrete order in a target system. Thus, such elements need to be converted and implemented in a backend system.

### Technical Mapping

The last task within our approach is given by a technical translation to actually generate ready-to-use pathways. Basically we have to compile one formalism into another and this kind of problem has been successfully solved over decades through methods of theoretical computer science. So we can use long time approved methods at this point. Coming from the HL7 specific XML structure of the meta-model, there are at least three different translation schemata to mention:

-  XML to other XML syntaxes
-  XML to a relational data model
-  XML to other formalisms

The translation can be realized using a transformational language like XSLT (XSL Transformation), if the target formalism is XML specific either. Additionally, there exist several methods to map XML data into relational tables, cf. [24].

### Exemplary deployment process for a breast carcinoma pathway and the path module DOC.Path

The deployment process outlined above will now be clarified by one concrete example. A simplified pathway for the treatment of a breast carcinoma is represented by a HL7 specific XML notation and consists of an inclusion diagnosis (malignant neoplasm of lower-inner quadrant of breast) and a treatment phase (preoperative day), which contains the patients admission (encounter). Following, this pathway will be integrated into the path module DOC.Path of the HIS iMedOne.

In a first step the XML document is parsed to select all specified path elements. To use this data during the deployment process, it is transformed into an ontological description. The template for this translation is given through the ontological specification of the meta-model, where all entities and relationships of a guideline-compliant pathway are defined. Based on this input, an instance model for the breast carcinoma pathway is generated.

**Figure 4:**
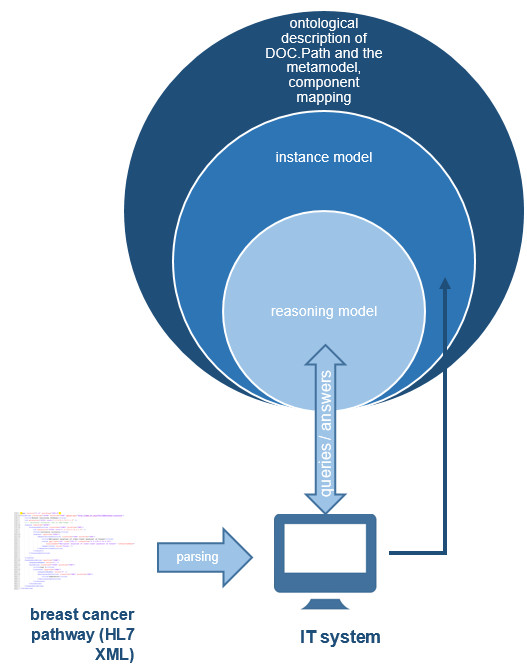
**Deployment process.** Illustrates the different ontological descriptions, which are used during deployment process to reason about the possible representation forms within a target system.

To enable the IT system to reason about the possible representations of the three pathway elements within DOC.Path, a reasoning model is created. It merges the instance model, the ontological description of DOC.Path and the component mapping between DOC.Path and the meta-model and is responsible to answer questions about possible representations within DOC.Path. In order to translate the first path element (inclusion diagnosis), the system sends a query to the union model and asks which counterparts exists for that component. There is only one equivalent representation for the inclusion diagnosis within DOC.Path. Therefore that translation can be done automatically. The second path element (treatment phase) can be displayed within DOC.Path in several ways. First it can be placed in the dimension *phase*, second an optional element can be used to represent the treatment phase. The choice how to translate that path element is taken by the domain experts. So the system presents them the two different alternatives and the users choose one representation form by interacting with the system. According to this method, the whole translation process is realized and the domain experts are navigated through all the decisions to take. Figure 4 summarizes that process.

## Discussion

We evaluated the modelling and deployment processes by one concrete example. The breast carcinoma pathway was defined based on the guideline for the diagnosis and treatment of breast cancer [6] and was transformed into the iMedOne specific formalism. The encoding of the meta-model by Health Level 7 ensures a non-proprietary solution; the deployment process is not even required for HIS, which can import clinical pathways using a HL7 interface. In addition, the ontology engineering approach ensures a generic solution, which can be used for different target systems.

The realized IT support can be seen as a supplement to a path module in a HIS. We offer comprehensive functions to support the whole pathway development process, while HIS only provide the possibility to implement and enact already defined pathways. In addition, we enabled the domain experts in performing all necessary tasks in that approach without requiring detailed IT know how. Even the deployment process can be influenced and is no black box for them.

There are some research questions for future work. First, the whole approach will be evaluated in a German hospital to analyse, whether the methodological approach and the developed IT system fit to the domain experts’ needs. Second, the presented approach is currently a “one-way-strategy”, which translates existing guidelines into executable pathways. For future work, a cycle model needs to be realized to reflect already existing pathways and lived processes in the hospitals. A measurement of the processes can indicate whether a pathway meets the recommendations of existing guidelines.

## Conclusions

We presented a model-driven approach, which supports hospitals and other healthcare facilities in developing guideline-compliant pathways. As a result, the latest scientific findings can be transferred into clinical routine. In contrast to related work, our goal is to support the entire lifecycle of guideline-compliant pathways by one IT system including the definition and deployment of the pathways in different target systems.
